# Supplementary material for: Host-associated microbe PCR (hamPCR) enables convenient measurement of both microbial load and community composition
Source: eLife. 2021 Jul 22;10:e66186. doi: 10.7554/eLife.66186 (PMC8387020; doi:10.7554/eLife.66186)
Supplement: Transparent reporting form [file elife-66186-transrepform.docx]

***eLife’s* transparent reporting form**

We encourage authors to provide detailed information *within their submission* to facilitate the interpretation and replication of experiments. Authors can upload supporting documentation to indicate the use of appropriate reporting guidelines for health-related research (see [EQUATOR Network](http://www.equator-network.org/%20)), life science research (see the [BioSharing Information Resource](https://biosharing.org/)), or the [ARRIVE guidelines](http://www.plosbiology.org/article/info:doi/10.1371/journal.pbio.1000412) for reporting work involving animal research. Where applicable, authors should refer to any relevant reporting standards documents in this form.

If you have any questions, please consult our Journal Policies and/or contact us: [editorial@elifesciences.org](mailto:editorial@elifesciences.org).

**Sample-size estimation**

- You should state whether an appropriate sample size was computed when the study was being designed
- You should state the statistical method of sample size computation and any required assumptions
- If no explicit power analysis was used, you should describe how you decided what sample (replicate) size (number) to use

Please outline where this information can be found within the submission (e.g., sections or figure legends), or explain why this information doesn’t apply to your submission:

Sample sizes were not computed in advance

**Replicates**

- You should report how often each experiment was performed
- You should include a definition of biological versus technical replication
- The data obtained should be provided and sufficient information should be provided to indicate the number of independent biological and/or technical replicates
- If you encountered any outliers, you should describe how these were handled
- Criteria for exclusion/inclusion of data should be clearly stated
- High-throughput sequence data should be uploaded before submission, with a private link for reviewers provided (these are available from both GEO and ArrayExpress)

Please outline where this information can be found within the submission (e.g., sections or figure legends), or explain why this information doesn’t apply to your submission:

In this work, we define a technical replicate as independently produced sequencing libraries that originated from the same biological material and DNA extraction (same theoretical underlying distribution of host and microbial molecules). We define a biological replicate as coming from independent biological material.

In Figure 5a-c and Figure 6, we use biological replicates from independent pepper and maize leaf hole punches. Each experiment with biological replication was performed only once. We consider this sufficient because we are focused on technical performance of our method rather than the reproducibility of biological phenomena.

**Statistical reporting**

- Statistical analysis methods should be described and justified
- Raw data should be presented in figures whenever informative to do so (typically when N per group is less than 10)
- For each experiment, you should identify the statistical tests used, exact values of N, definitions of center, methods of multiple test correction, and dispersion and precision measures (e.g., mean, median, SD, SEM, confidence intervals; and, for the major substantive results, a measure of effect size (e.g., Pearson's r, Cohen's d)
- Report exact p-values wherever possible alongside the summary statistics and 95% confidence intervals. These should be reported for all key questions and not only when the p-value is less than 0.05.

Please outline where this information can be found within the submission (e.g., sections or figure legends), or explain why this information doesn’t apply to your submission:

In Figures 2 and 3, Kolmogorov-Smirnov tests to judge the similarity of amplicon sequence variant (ASV) distributions were carried out using programming language R using the function “ks.test()”. Only amplicon sequence variants (ASVs) with a minimum relative abundance of 0.05% were compared. ASVs meeting this threshold were fourth-root transformed prior to using the test. Insignificant test results (*p* > 0.05) signify distributions that are not different from each other.

In Figure 6b, the comparison of total bacterial loads of bacterial classes between 0, 2, and 4 dpi was done with a Mann-Whitney U-test in R using the function “wilcox.test()”.

For all correlations reported, the coeffetion of determination (square of Pearson’s r) is shown directly in the figure.

(For large datasets, or papers with a very large number of statistical tests, you may upload a single table file with tests, Ns, etc., with reference to sections in the manuscript.)

**Group allocation**

- Indicate how samples were allocated into experimental groups (in the case of clinical studies, please specify allocation to treatment method); if randomization was used, please also state if restricted randomization was applied
- Indicate if masking was used during group allocation, data collection and/or data analysis

Please outline where this information can be found within the submission (e.g., sections or figure legends), or explain why this information doesn’t apply to your submission:

N/A

**Additional data files (“source data”)**

- We encourage you to upload relevant additional data files, such as numerical data that are represented as a graph in a figure, or as a summary table
- Where provided, these should be in the most useful format, and they can be uploaded as “Source data” files linked to a main figure or table
- Include model definition files including the full list of parameters used
- Include code used for data analysis (e.g., R, MatLab)
- Avoid stating that data files are “available upon request”

Please indicate the figures or tables for which source data files have been provided:

N/A
